# Ultrasound irradiation in the presence of microbubbles may enhance the antitumor effect of chemotherapeutic agents against bladder cancer

**DOI:** 10.7150/jca.100846

**Published:** 2025-01-01

**Authors:** Takehiro Suzuki, Takuma Sato, Ariunbuyan Sukhbaatar, Maya Sakamoto, Shiro Mori, Tetsuya Kodama, Akihiro Ito

**Affiliations:** 1 Department of Urology, Tohoku University Graduate School of Medicine, Seiryo-machi, Aoba-ku, Sendai, Miyagi 980-8574, Japan.; 2 Division of Oral and Maxillofacial Oncology and Surgical Sciences, Graduate School of Dentistry, Tohoku University, Seiryo-machi, Aoba-ku, Sendai, Miyagi 980-8575, Japan.; 3 Department of Dental Informatics and Radiology, Graduate School of Dentistry, Tohoku University, Seiryo-machi, Aoba-ku, Sendai, Miyagi 980-8575, Japan.; 4 Graduate School of Biomedical Engineering, Tohoku University, Seiryo-machi, Aoba-ku, Sendai, Miyagi 980-8575, Japan.

**Keywords:** bladder cancer, drug delivery, intravesical instillation of chemotherapy, sonoporation

## Abstract

Intravesical instillation of chemotherapy has been performed to reduce the risk of intravesical recurrence of bladder cancer. However, its antitumor effect is not necessarily sufficient, which may be partially due to inadequate delivery of intravesically administered chemotherapeutic agents to bladder tumors. Ultrasound irradiation to target tissues in the presence of microbubbles is a technique to transiently enhance cell membrane permeability and achieve efficient drug delivery to the desired sites without damage to non-target areas; this technique has been used in chemotherapy, immunotherapy, gene therapy, and radiotherapy for the treatment of various cancers. However, the effectiveness of combining intravesical instillation of chemotherapy and this strategy for the treatment of bladder cancer has not been fully investigated.

This report shows that mitomycin C combined with ultrasound and microbubbles has a higher antitumor effect than mitomycin C alone against mouse bladder cancer cells. Next, the antitumor effect of intravesical instillation of chemotherapy combined with ultrasound and microbubbles was demonstrated using an orthotopic mouse bladder cancer model. *In vivo* experiments showed that ultrasound irradiation in the presence of microbubbles enhanced the local delivery of fluorescent molecules and had the potential to enhance the antitumor effect of intravesical instillation of chemotherapy without visible damage to the surrounding normal tissues. The results of the present study demonstrate that intravesical chemotherapy combined with ultrasound and microbubbles is potentially a safe and effective treatment for bladder cancer.

## Introduction

Non-muscle-invasive bladder cancer (NMIBC) accounts for 70% of newly diagnosed bladder cancer cases, and the standard treatment for NMIBC is transurethral resection of the bladder tumor (TURBT) 1. However, TURBT alone is known to inadequately prevent intravesical recurrence and disease progression 2. In clinical practice, immediate post operative instillation of chemotherapy (IPIC) and additional adjuvant intravesical chemotherapy have been widely used after initial TURBT, and they contribute to reducing the risk of recurrence 3-5. A systematic review and individual patient data meta-analysis showed that IPIC reduced the 5-year recurrence rate by 14% 6. A meta-analysis showed that additional adjuvant intravesical chemotherapy reduced the 1-year recurrence rate by 38% 7. Given global concerns of a bacillus Calmette-Guérin (BCG) shortage, intravesical chemotherapy is expected to produce a high antitumor effect 8. However, in clinical practice, intravesical chemotherapy has not played a role comparable to intravesical BCG therapy. Although IPIC contributes to reducing recurrence risk in patients with NMIBC overall, the strategy has been reported not to reduce the risk of recurrence in patients with a prior recurrence rate of >1 recurrence/year and European Organization for Research and Treatment of Cancer (EORTC) recurrence score ≥5. In addition, it may not provide clinical benefit for patients with high-risk and multiple tumors 9. We hypothesized that, although intravesically administered chemotherapeutic agents remain in the bladder at a high concentration for a long period of time, they are not delivered efficiently to target tissues (tumors in the bladder), which may be responsible for their insufficient antitumor effect. There are several techniques to improve the anti-tumor effect of intravesical chemotherapy, including electro-motive drug administration and chemohyperthermia 10, 11. These strategies require dedicated devices, and in clinical studies in which oncological outcomes were compared between their use in intravesical chemotherapy and conventional intravesical therapy, the clinical advantages of the former therapies over conventional therapy have been limited 10, 12, 13. Ultrasound (US) irradiation in the presence of microbubbles (MBs) induces transient and reversible membrane poration of target cells, so-called sonoporation, which can enhance the delivery of exogenous molecules to the target tissues; it may also improve the antitumor activity of chemotherapeutic drugs against target neoplastic cells without visible damage to the surrounding normal tissues 14-16. Sonazoid consists of perfluorobutane gas stabilized with lipid membranes, with a particle size of 2.3-2.9 μm 17. Sonoporation is a safe and promising technique for enhancing drug delivery efficiency, and its usefulness has been proven for various types of malignancies; in addition, attempts have been made to apply this technique to chemotherapy, immunotherapy, gene therapy, and radiotherapy for the treatment of various types of cancer 18-25. In our previous study, we showed that sonoporation enhanced the delivery of exogenous materials to the tissues in the bladder 26. This study aimed to investigate antitumor efficiency of anticancer agents combined with US and MBs, using agents that are available in clinical practice (mitomycin C [MMC] and Sonazoid), against bladder cancer cells* in vitro* and determine whether their combination can enhance the antitumor effect of intravesical instillation of chemotherapy in an experimental mouse bladder cancer model.

## Materials and Methods

### Cell preparation

MBT-2 (bladder cancer) cells were established from C3H/HeN mice and obtained from the RIKEN BioResource Research Center. MBT-2 cells were maintained in RPMI-1640 medium (Sigma-Aldrich, St Louis, MO, USA) supplemented with 10% heat-inactivated fetal bovine serum (HyClone Laboratories Inc., South Logan, UT, USA) and 1% L-glutamine-penicillin-streptomycin (Sigma-Aldrich). Cells were incubated at 37 °C in a mixture of 5% carbon dioxide and 95% air. Before *in vitro* and *in vivo* experiments, the absence of mycoplasma contamination in the cell cultures was ensured using a MycoAlert Mycoplasma Detection Kit (Lonza Rockland, Inc., Rockland, ME, USA), according to the manufacturer's protocol.

### Preparation of MBs

Sonazoid (Daiichi Sankyo, Tokyo, Japan), which contains perfluorobutane (C_4_F_10_) gas, was used as MBs, and it was prepared according to the manufacturer's protocol and used within 2 h of preparation.

### Ultrasound irradiation

A flat, disc-shaped, 1.0-MHz US transducer with a diameter of 12 mm (BFC Applications, Fujisawa, Japan) was used for irradiation. US exposures were carried out in a test chamber filled with tap water heated to 38 °C. For the* in vitro* experiments, the US parameters were as follows: intensity, 1.0 W/cm^2^ (peak negative pressure, 0.12 MPa); duty cycle, 50%; number of pulses, 2000; pulse repetition frequency, 250 Hz; and exposure time, 10 s. For the* in vivo* experiments, the corresponding parameters were 8.0 W/cm^2^ (0.35 MPa), 20%, 200, 1000 Hz, and 60 s.

### *In vitro* study of cell viability assay

Cell viability was measured by a 3-(4,5-dimethylthiazol-2-yl)-2,5-diphenyltetrazolium bromide (MTT) assay. MBT-2 cells were trypsinized with trypsin-EDTA (FUJIFILM Wako Pure Chemical Corporation, Osaka, Japan) and suspended at a concentration of 1.0 × 10^4^ cells/mL in the same medium used for cell preparation (see above). Subsequently, 500 μL of samples were aliquoted into 48-well plates and incubated for 24 h at 37 °C in a mixture of 5% carbon dioxide and 95% air. After the supernatant in each well was removed, for the control group, 90 μL of medium and 10 μL of phosphate buffered saline (PBS; Sigma-Aldrich) were added; for the MMC group, 90 μL of medium and 10 μL of MMC in PBS (with final MMC concentrations of 50 or 100 μM) were added; for the US + MB group, 90 μL of medium and 10 μL of Sonazoid were added; and for the US + MB + MMC group, 80 μL of medium and 10 μL of MMC in PBS and 10 μL of Sonazoid were added, with the final concentration of MMC being 50 or 100 μM. In each group, samples were positioned 1mm above a 12-mm-diameter US transducer immersed in tap water (degassed and heated to 38 °C), and then samples from the US + MB and US + MB + MMC groups were exposed to US (10 s; 1.0 W/cm²). One hour later, the supernatant of each well was removed and washed twice with 300 μL of PBS. Subsequently, 500 μL of the same medium used for cell preparation were added, and the samples were incubated. Then, 48 h later, cell viability was determined by an MTT assay, as described previously 27. Each experiment was performed using five samples. Normalized cell viability was obtained by dividing the value for each treated sample by the mean of the control samples.

### *In vitro* study by the cell migration assay

Cell migration was measured by the wound healing assay. A comparison of control, US + MB, MMC, and US + MB + MMC groups (*n* = 5 for each) was performed. Immediately after treatment as described above (cell viability assay), the cell monolayer was scratched by a sterile 1000-μL pipette tip (which was held vertically to go across each well), observed under a microscope, and an image was taken with a digital camera to measure the gap. The detached cells were removed by washing with 300μL of PBS twice, and subsequently, 500μL of the same medium (see above) were added and incubated at 37 °C for 48 h (in a mixture of 5% carbon dioxide and 95% air). Wound closure area (%) was calculated by (A0 - A1)/A0 × 100, where A0 represents the area of the initial photographed wound, and A1 represents the photographed remaining area of the wound 48 h later. The normalized wound closure area was obtained by dividing the value for each treated sample by the mean of the control samples.

### *In vitro* study by the cell invasion assay

For invasion assays, a Transwell chamber (3422, Corning, Corning, NY, USA) with 24-well plate cell culture inserts (pore size 8 µm) was used. First, diluted Matrigel (Corning) was placed on the upper side of the chamber, and the chambers were placed in an incubator at 37 °C for at least 12 h to solidify the Matrigel solution. Then, samples from the control, US + MB, MMC, and US + MB + MMC groups (*n* = 5 each) were compared. Immediately after treatment as described above (cell viability assay), the cells were detached with trypsin-EDTA, collected in 250 μL of serum-free RPMI medium, and seeded in the upper chamber. Then, 750 μL of RPMI with 10% FBS were added to the lower chamber and incubated at 37 °C for 96 h in a mixture of 5% carbon dioxide and 95% air. After 96 h, non-migrated cells remaining on the filter upside of the chambers were wiped away with a swab. Migrated cells that invaded into the lower chamber were fixed with 70% ethanol, stained with HE, photographed and counted. Normalized invaded cell values were obtained by dividing the value for each treated sample by the mean of the control samples.

### *In vitro* study by quantitative RT-PCR analysis

To investigate the possible mechanisms underlying the antitumor action of MMC combined with US and MBs against MBT-2 cells, the expression levels of apoptosis-related genes were quantified in the control, MMC, US + MB and US + MB + MMC groups (*n* = 6 for each). The US intensity used was 1.0 W/cm^2^, and the MMC concentration was 50 μM. Samples were treated as described for measurement of cell viability and subsequently incubated for 6 h, and then total RNA was extracted from each sample with the FastGene RNA Premium Kit (NIPPON Genetics Co., Ltd., Tokyo, Japan) and cDNA was synthesized using a High-Capacity cDNA Reverse Transcription Kit (Applied Biosystems, Foster City, CA, USA) according to the manufacturer's protocol. Then, real-time quantitative PCR analysis was performed with the Thermal Cycler Dice Real Time System II (Takara Bio, Shiga, Japan). The relative quantification method (2^-ΔΔCT^) was used to determine relative mRNA expression, using β-actin as the reference. The expression levels of BAX, bcl-2, p53, caspase3, caspase8, and caspase9 genes were normalized against a reference gene (β-actin), and fold change was determined relative to the control. The PCR primers used were (forward, reverse): murine (m)-β-actin, 5'-GATCATTGCTCCTCCTGAGC-3', 5'-ACATCTGCTGGAAGGTGGAC-3'; m-BAX, 5'-ATGGAGCTGCAGAGGATGATTG-3', 5'-TGATCAGCTCGGGCACTTTAG-3'; m-bcl-2, 5'-CCACAGCAGCAGTTTGGATG-3', 5'-AAACTCATCGCCTGCCTCTC-3'; m-p53, 5'-GCGTAAACGCTTCGAGATGTT-3', 5'-TTTTTATGGCGGGAAGTAGACTG-3'; m-caspase3, 5'-TGAAGGGGTCATTTATGGGACA-3', 5'-CCAGTCAGACTCCGGCAGTA-3'; m-caspase8, 5'-CAACTTCCTAGACTGCAACCG-3', 5'-TCCAACTCGCTCACTTCTTCT-3'; and m-caspase9, 5'-TCCTGGTACATCGAGACCTTG-3', 5'-AAGTCCCTTTCGCAGAAACAG-3'.

### Bladder cancer model

A 24-gauge catheter with a sterile syringe was inserted through the urethra into the bladder of 24 female C3H/HeN mice aged 11-12 weeks (CLEA Japan Inc., Tokyo, Japan) under general anesthesia using 2% isoflurane (Pfizer Japan Inc., Tokyo, Japan). After elimination of urine, a bolus of 100 µL of trypsin-EDTA (FUJIFILM Wako Pure Chemical Corporation) was infused into the mice's bladders, retained for 20 min, and then the trypsin was drained. Thereafter, mucosa on the posterior wall or dome of the bladder was injured using a 24-gauge intravascular needle (Terumo, Tokyo, Japan). This was performed under US guidance, using a high-frequency US (HFUS) imaging system (Vevo 770; VisualSonics Inc., Toronto, Canada). MBT-2 cells were suspended in PBS, and 100 μL (concentration: 5.0 × 10^6^ cells/mL) of cell suspension were injected into the bladder. The urethra of the mice was ligated with 4-0 monofilament to retain the cell suspension in the bladder. After 90 min, ligation was released. The day of inoculation was defined as day 0.

### Delivery of fluorescent molecules

An* in vivo* experiment to investigate whether US irradiation combined with MBs could enhance target delivery of exogenous molecules into the tumor cells in the bladder was carried out using the mouse bladder cancer model described above. For the control sample, a total volume of 100 μL, containing 10 μL of 10 mM TOTO-3 (T-3604; Molecular Probes, Eugene, OR, USA; molecular weight, 1355; absorption, 642 nm; emission, 660 nm), 25 μL of Sonazoid, and 65 μL of PBS were injected into the bladder of the experimental mouse model. Then, the mouse was immersed in the test chamber (filled with tap water heated to 38 °C), and US irradiation was not performed. For the US treatment sample, a total of 100 μL of solution, the same as above, were injected into the bladder of the experimental mouse. After HFUS imaging of the tumor in the bladder, the areas to be irradiated were determined. The mouse was immersed in the test chamber described above, positioned with its surfaces at a distance of 25 mm from the 12-mm US transducer. Subsequently, the bladder containing tumor was irradiated by US through the abdominal wall (duty ratio, 20%; exposure time, 60 s × 3 times; and number of cycles in the pulse, 200). After US irradiation, collapse of MBs was monitored using the HFUS imaging system. The anesthetized mice were euthanized, and whole bladders were extracted, embedded in optimum cutting temperature compound (Tissue-Tek, Torrance, CA, USA), and rapidly frozen in a bath of liquid nitrogen.

### Imaging of the delivery of fluorescent molecules and analysis

The samples were sectioned at 10 µm using a cryostat microtome (CM1950; Leica Biosystems, Nussloch, Germany). Nuclei were counterstained with 100 ng/mL of 4′,6-diamidino-2-phenylindole (DAPI) staining solution (Sigma-Aldrich) at room temperature. Images showing DAPI (excitation, 404 nm; emission, 425 to 475 nm) and TOTO-3 (excitation, 640 nm; emission, 663 to 738 nm) fluorescence were captured using a laser scanning confocal microscope (A1-SIM; Nikon, Tokyo, Japan). For histological analysis, adjacent sections were stained with HE using standard procedures. To visualize the distribution of TOTO-3 taken into the target tissues, the red signals were converted into 8-bit (grayscale) values, and signal intensity in the region of interest (ROI; 630 μm × 630 μm) was evaluated using ImageJ software, as previously described 28-30.

### *In vivo* ultrasound treatment

Twenty-four female C3H/HeN mice were used for the *in vivo* experiments. The experimental animals were divided into four groups as follows: control (*n* = 6), MMC (*n* = 6), US + MB (*n* = 6), and US + MB + MMC (*n* = 6). On day 2 (with the day of inoculation defined as day 0), HFUS imaging of the bladder was performed, and when tumor was detected in the bladder of experimental mice, they were considered eligible for subsequent experiments. In each group, treatment was carried out on day 2. For the control group, 100 μL of PBS were injected into the bladder of experimental mice using a 24-gauge catheter with a sterile syringe. For the MMC group, 5 μg/body weight MMC in 100 μL of PBS were injected. For the US + MB group, 25 μL of Sonazoid mixed with 75 μL of PBS were injected. For the US + MB + MMC group, 5 μg/body weight MMC in 75 μL of PBS mixed with 25 μL of Sonazoid were injected. After administration of the aforementioned agents, in the US + MB and US + MB + MMC groups, US irradiation was performed in the same way as described in the fluorescent molecules delivery experiment.

### Monitoring and measurement of bladder tumor volume

Time-dependent changes of bladder tumor volume in each experimental mouse were investigated. Each mouse was anesthetized with 2.0% isoflurane in oxygen. Bladder tumors were visualized with the HFUS imaging system with a 55-MHz center-frequency transducer (RMV-708), on days 2, 4, 6, 8 and 10. Consecutive 2-D images (slice thickness, 100 µm) of the bladder tumor were collected, and then the boundary of the tumor in each image was traced manually on the 2-D images. These images were automatically reconstructed as 3-D images using quantification software (VisualSonics) to obtain bladder tumor volume. Bladder tumor volumes were normalized to values obtained on day 2.

### Histological analysis

On day 10, euthanasia by cervical dislocation under inhalation anesthesia was carried out. Ten percent formalin (FUJIFILM Wako Pure Chemical Corporation) was administered via the urethra into the bladder, the urethra was ligated, and then the bladder was removed. Subsequently the bladder was fixed by formalin for 4 days, dehydrated with ethanol for 2 days, embedded in paraffin, and cut into 2.5- μm-thick serial sections. After staining the sections with HE, the tumor was observed under a microscope (BX-51, Olympus, Tokyo, Japan).

### Statistical analysis

Data are expressed as mean ± standard error of the mean (SEM) values. Comparisons between groups were made using one-way analysis of variance (ANOVA) and the Tukey-Kramer test. The differences were considered significant at *P* < 0.05. Statistical analysis was performed using GraphPad Prism 8 (GraphPad Software, Inc.).

## Results

### *In vitro* US treatment

**Figure 1:**
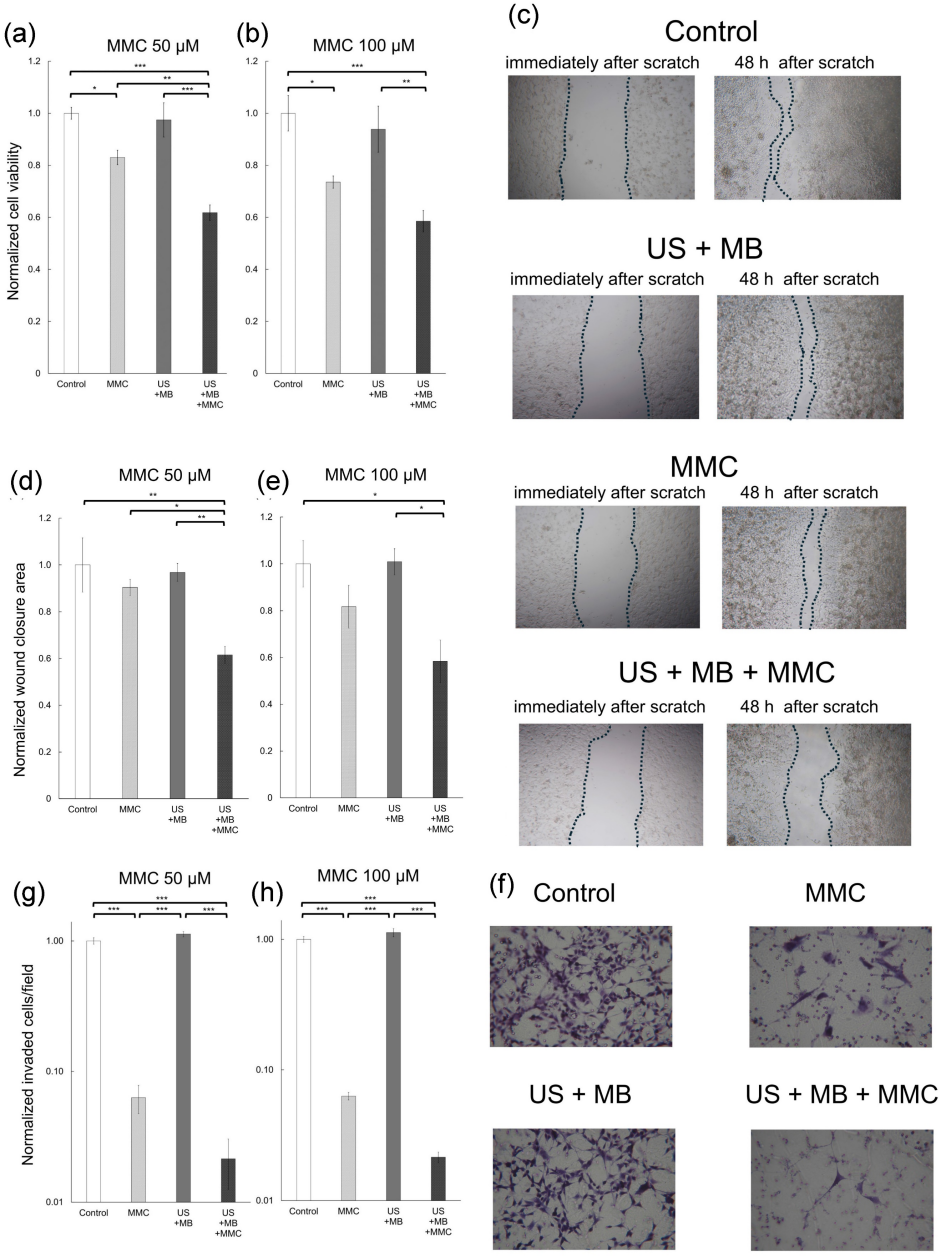
*In vitro* experiments of cell viability, cell migration, and cell invasion. To obtain normalized cell viability, normalized wound closure area (migration), and normalized invaded cells/field (invasion), the value in each group was divided by the mean of the control samples. Panels (a) and (b) represent normalized cell viability of MBT-2 cells measured with the 3-(4,5-dimethylthiazol-2-yl)-2,5-diphenyltetrazolium bromide (MTT) assay, at mitomycin C (MMC) concentrations of 50 μM and 100 μM. Panel (c) displays bright-field images of each group in the migration assay. Images from immediately and at 48 h after the scratch are shown. Panels (d) and (e) display the normalized wound closure area of MBT-2 cells at MMC concentrations of 50 μM and 100 μM. Panel (f) presents microscopic images of each group in the invasion assay. Panels (g) and (h) present normalized invaded cells/field of MBT-2 cells at MMC concentrations of 50 μM and 100 μM. Significant differences in normalized cell viability and normalized wound closure area are found between the MMC and US + MB + MMC groups at MMC concentration of 50 μM. Mean ± SEM values are shown. *P < 0.05. **P < 0.01. ***P < 0.001.

To assess whether the antitumor effect of MMC was enhanced by US and MBs, cell viability in each experimental group was analyzed with the MTT assay (Fig. 1a, b). At an MMC concentration of 50 μM, normalized cell viability was significantly lower in the US + MB + MMC group than in the other three groups. At an MMC concentration of 100 μM, normalized cell viability was significantly lower in the US + MB + MMC group than in the control and US + MB groups. There was a trend towards lower normalized cell viability in the US + MB + MMC group, compared with that in the MMC group, but with no significant difference. To examine whether MMC combined with US and MBs affects migration and invasion of MBT-2 cells, scratch and Matrigel invasion assays were performed (Fig. 1c-h). At an MMC concentration of 50 μM, the normalized wound closure area was significantly smaller in the US + MB + MMC group than in the other three groups. At an MMC concentration of 100 μM, the normalized wound closure area was significantly smaller in the US + MB + MMC group than in the control and US + MB groups. There was a trend towards smaller wound closure area in the US + MB + MMC group than in the MMC group, but no significant difference was demonstrated. At MMC concentrations of 50 and 100 μM, the value of the normalized invaded cells/field was significantly less in the US + MB + MMC group than in the control and US + MB groups. In addition, the value of the normalized invaded cells/field tended to be lower in the US + MB + MMC group than in the MMC group.

### Measurements of the expression levels of apoptosis-related genes

**Figure 2:**
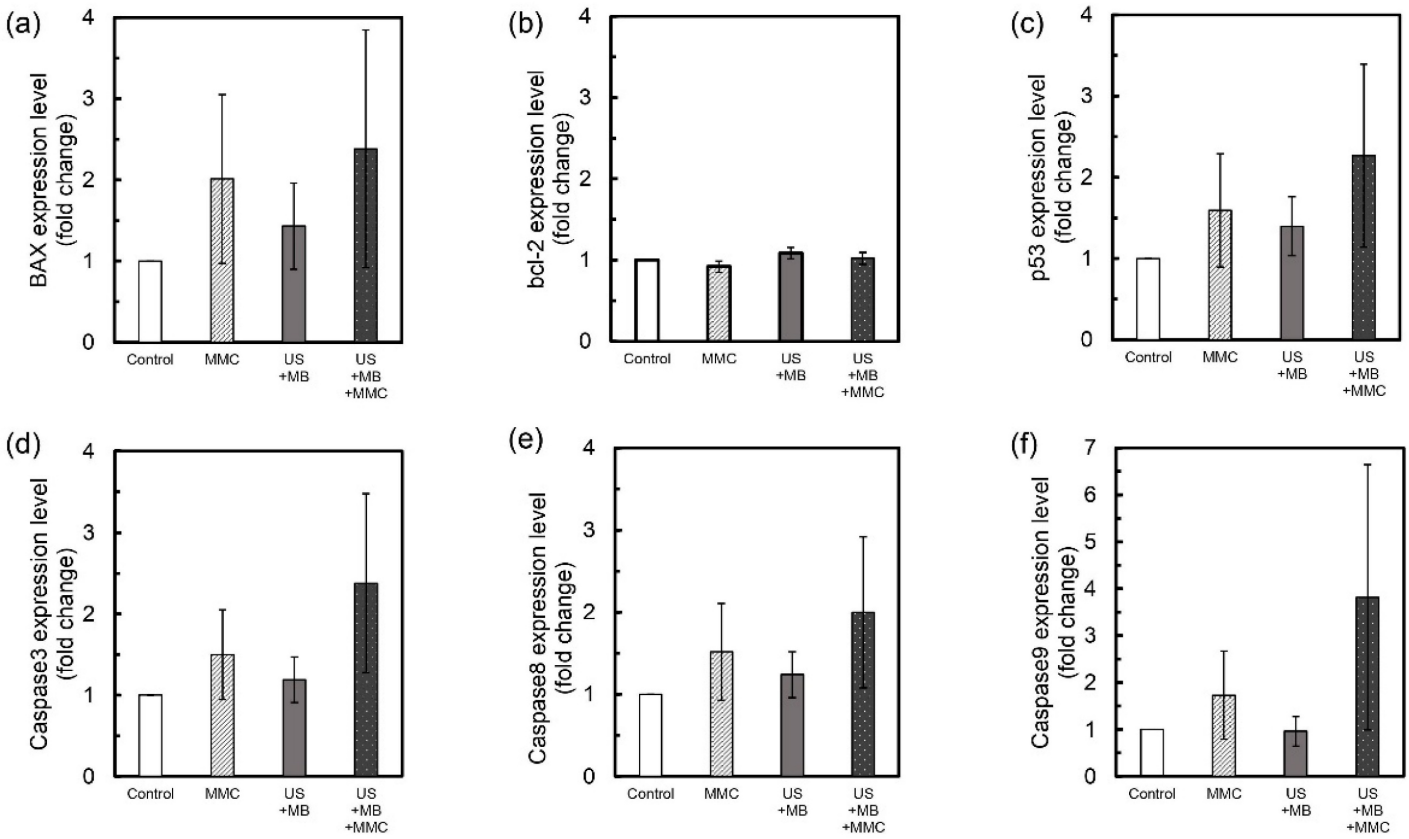
Expression levels of apoptosis-related genes. The mean fold-change in the expression level of each gene, relative to the control group, was measured for the US + MB, MMC, and US + MB + MMC groups (*n* = 6 for each group). Expression levels of BAX (a), bcl-2 (b), p53 (c), caspase3 (d), caspase8 (e) and caspase9 (f) genes in MBT-2 cells are shown. There is a trend towards higher expression of BAX, p53, caspase3, caspase8, and caspase9 genes in the US + MB + MMC group compared with the other 3 groups. Mean ± SEM values are shown.

To determine the underlying mechanism of antitumor activity of MMC combined with US and MBs, expression levels of 6 apoptosis-related genes (BAX, bcl-2, p53, caspase3, caspase8, caspase9) were analyzed. There was a trend towards higher expression levels of BAX, p53, caspase3, caspase8, and caspase9 genes in the US + MB + MMC group than in the other groups, but no significant differences were demonstrated (Fig. 2a, c-f). Only a small change in expression levels of bcl-2 gene was found among the groups (Fig. 2b).

### Localization of fluorescent molecules to bladder tumors on histological analysis

**Figure 3:**
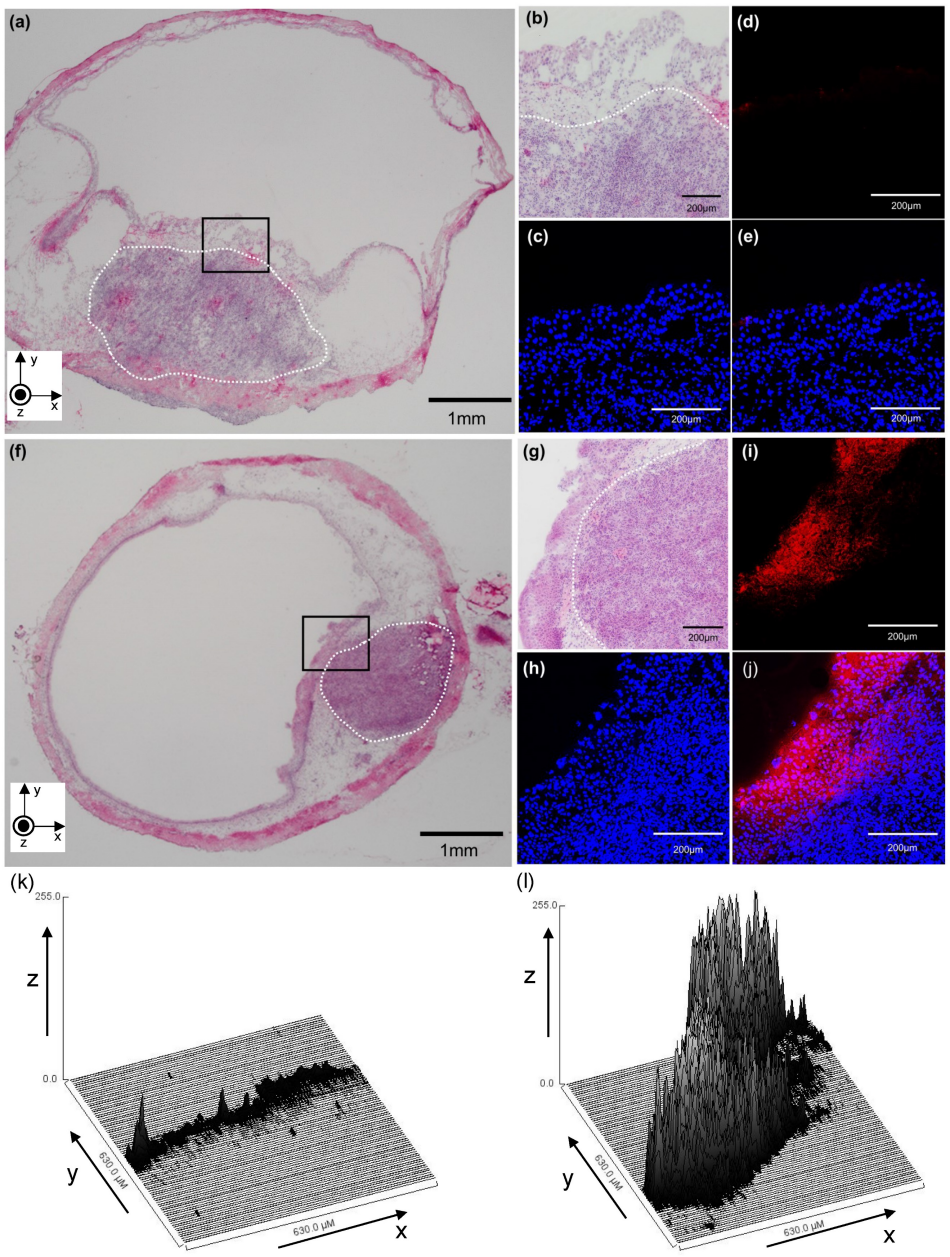
A representative confocal image (cross-section) of a bladder in which TOTO-3 fluorescence molecules were delivered using microbubbles (MBs) and ultrasound (US). (a-e) MBs and TOTO-3 without US irradiation. (f-j) MBs and TOTO-3 with US irradiation. (a) A section stained with hematoxylin and eosin (HE). The white dotted line indicates a carcinomatous lesion. (b) An HE-stained section of the bladder (black box) shown in (a) with higher magnification. (c) Nuclei of bladder cells (including MBT-2 cells) were stained with 4′,6-diamidino-2-phenylindole (DAPI). (d) TOTO-3 (red) fluorescence shows the location where the molecules are delivered. (e) Merged image. (f) HE-stained section. The white dotted line indicates a carcinomatous lesion. (g) An HE-stained section of the bladder (black box) shown in (f) with higher magnification. (h) DAPI staining. (i) TOTO-3 (red) fluorescence shows the location where the molecules are delivered. (j) Merged image. (k and l) The distribution of red signal intensity converted into 8-bit grayscale values is shown in a three-dimensional plot, where the x-axis and y-axis correspond to the x-y plane shown in (d) and (i), respectively, and the z-axis shows the mean grayscale intensity of the fluorescent molecule at each point in the region of interest.

To confirm target delivery of exogenous molecules by US irradiation in the presence of MBs, TOTO-3 fluorescence localization was analyzed by confocal microscopy. The nuclei were counterstained with DAPI. Figure 3 shows a cross-sectional confocal image of a bladder when TOTO-3 fluorescence molecules and MBs were injected into the bladder of this experimental mouse and US irradiation was subsequently performed. Figure 3a-e shows controls and Figure 3f-j shows treated samples. A hematoxylin and eosin (HE)-stained section of the bladder without the delivery sequence is shown in Figure 3a; Figure 3b displays higher magnification of the views highlighted in Figure 3a. Figure 3c-e shows the serial sections of the confocal image of Figure 3b. Nuclei of bladder cells were stained blue (Fig. 3c), and introduction of TOTO-3 fluorescence into the bladder tumors was not observed (Fig. 3d). As shown in Figure 3e, the merged image shows that the TOTO-3 fluorophore was not delivered into the bladder tumor. Figure 3f presents the HE-stained section of a bladder tumor that received US irradiation in the presence of MBs, and Figure 3g shows higher magnification of the views highlighted in Figure 3f. Figure 3h-j shows the serial sections of the confocal image of Figure 3g. Nuclei of bladder cells were stained blue (Fig. 3h), and the area into which TOTO-3 fluorophores had been delivered was visible (Fig. 3i).

The merged image indicated that TOTO-3 fluorophores were successfully delivered into the target cells/tissues (bladder tumor) using MBs with US irradiation; however, they were delivered only to the superficial areas of the bladder tumors (Fig. 3j). To better depict the distribution of TOTO-3 taken into target tissues, the red signal intensity of each of the images in Figure 3d,i is shown in three dimensions in Figure 3k,l, where the x-axis and y-axis correspond to the x-y plane shown in Figure 3d,i, with the z-axis indicating the intensity of the red signal, as previously described 28, 29. On grayscale intensity analysis, very little signal was detected in a sample from the experimental mouse that did not receive US irradiation (Fig. 3k). Increased signals were detected in a sample from the mouse that received US irradiation (Fig. 3l).

### *In vivo* intravesical instillation of MMC with US and MBs

**Figure 4:**
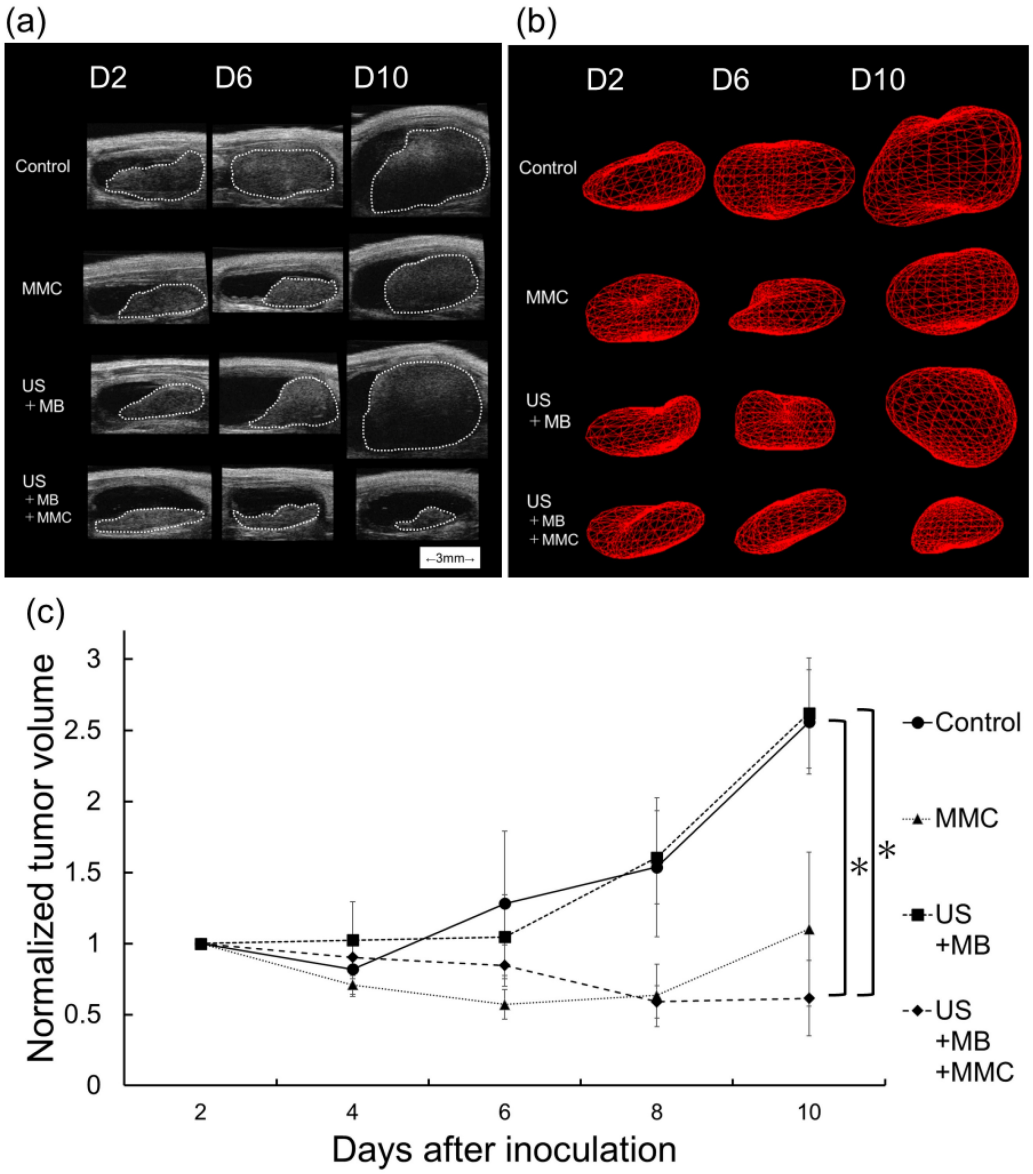
Evaluation of treatment efficacy in the experimental bladder cancer model. (a) Bladder imaging from the control, US + MB, MMC, and US + MB + MMC groups acquired using a high-frequency ultrasound imaging system for each group on days, 2, 6, and 10. The white dotted line indicates a carcinomatous lesion. (b) 3D reconstructed image of the bladder tumor. (c) Longitudinal analysis of normalized tumor volume from the four groups described above (*n* = 6 for each group). Values at different time points were normalized to those on day 2. Mean ± SEM values are shown. *P < 0.05.

The* in vitro* experiments for the MBT-2 cells described above showed that US and MBs enhanced the antitumor effect of MMC under a particular condition (MMC concentration of 50 μM). The antitumor activity exerted by combining intravesical instillation of MMC with US and MBs was evaluated *in vivo* using a mouse bladder cancer model. Figure 4a shows representative bladder imaging in each group acquired by an HFUS imaging system obtained on days 2, 6, and 10. The volume was calculated by 3D-reconstructed images from HFUS images of the bladder. Figure 4b shows representative 3D-reconstructed images of the bladder tumor in each group acquired on days 2, 6, and 10. As shown in Figure 4c, the normalized bladder tumor volume on day 10 was significantly lower in the US + MB + MMC group than in the control and US + MB groups; there was also a trend of the normalized volume of bladder tumor being lower in the US + MB + MMC group than in the MMC group.

### Histopathological analysis

**Figure 5:**
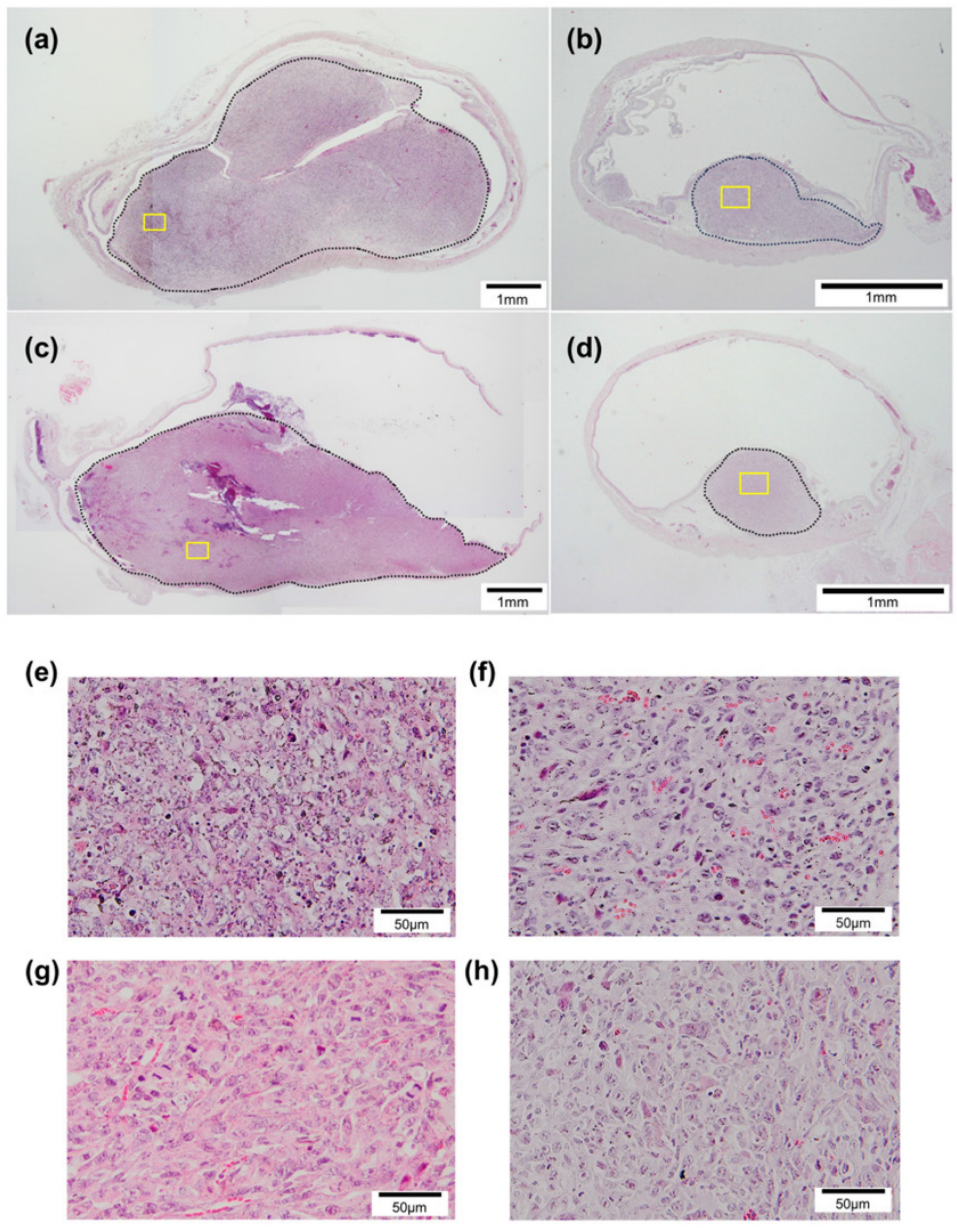
Histopathological analysis. Sections from each group stained with hematoxylin and eosin. (a) Control, (b) MMC, (c) US + MB, and (d) US + MB + MMC. Panels (e), (f), (g), and (h) show representative microscopic images with higher magnification of panels (a), (b), (c), and (d) (yellow boxes), respectively. The black dotted lines indicate carcinomatous lesions. In the control and US + MB groups, widespread cancerous lesions are observed, whereas relatively small lesions are observed in sections from the MMC and US + MB + MMC groups.

To monitor the antitumor effects of each treatment, histological analysis was performed. Figure 5 shows representative HE-stained sections of the bladder acquired on day 10 in each group. Tumors protruding into the bladder lumen were observed in all groups (Fig. 5a-d). In the control and US + MB groups, the tumors grew to occupy the intravesical lumen of the bladder (Fig. 5a, c). In the MMC and US + MB + MMC groups, a relatively small tumor was found in the bladder (Fig. 5b, d). There was no visible damage to the normal tissue surrounding the tumor cells in either group.

## Discussion

US irradiation in the presence of nano-microbubbles (sonoporation) is a non-invasive technique to deliver therapeutic compounds into specific target cells/tissues 26. It has been used as a strategy to enhance the antitumor effects of conventional therapies for malignancies including chemotherapy, radiotherapy, and immunotherapy 24, 25, 31. In addition, attempts have been made to use the technique for the treatment of non-malignant diseases (e.g., urinary tract infections) 32. In the present study, intravesical instillation of chemotherapy combined with US and MBs (sonoporation) was used in the treatment of bladder cancer.

In the present study, the effects on cell viability, migration, and invasion of bladder cancer cells (MBT-2) when anticancer drugs and MBs were administered concurrently, followed by US irradiation, were investigated. In our previous reports, chemotherapeutic agents combined with US and MBs significantly reduced cell viability of various types of malignant cells, compared with chemotherapeutic agents alone; however, enhancement of the antitumor effect by US and MBs was shown under certain conditions (e.g., under specific drug concentrations or US parameters) 33. In the present study, Sonazoid was used as MBs because anticancer drugs combined with US and Sonazoid exerted a higher antitumor effect than anticancer drugs alone in a previous preclinical study 23. In addition, at present, Sonazoid is the only source of MBs approved for clinical use and covered by health insurance in Japan.

In the present study, a significant difference in normalized cell viability between the MMC and US + MB + MMC groups was demonstrated only when the MMC concentration was 50 μM (Fig. 1a, b), suggesting that the sonoporation method did not necessarily enhance the antitumor action of chemotherapeutic drugs under any conditions. Cancer cell migration and invasion are deeply associated with recurrence, metastasis, and microvascular invasion of malignancies 34. In a recent report, a combination of anticancer drugs with US and MBs inhibited cell migration in prostate cancer cells 35. The results of cell invasion and migration assays showed that MMC combined with US and MBs may suppress migration and invasion of bladder cancer cells. However, a significant difference in normalized wound closure area between the MMC and US + MB + MMC groups was found under the particular condition of an MMC concentration of 50 μM, and no significant difference was found in normalized invaded cells/field between the two groups (Fig. 1c-h). Previous reports have shown that there are optimal conditions under which chemotherapeutic agents and US combined with MBs act synergistically and achieve an enhanced antitumor effect. The optimal concentrations of chemotherapeutic agents, when their antitumor activity is significantly enhanced by US combined with MBs, have been reported to be neither too high nor too low, and they also depend on the types of cell lines used. However, underlying mechanisms that explain why high antitumor activity is shown only in these particular conditions have not been fully identified. In addition, not only the concentration of chemotherapeutic agents, but also US parameters (e.g., intensity, frequency, duty ratio, and exposure time) have been associated with antitumor activity of chemotherapeutic agents combined with US and MBs. The US parameters in the present study were determined based on our previous studies 26, 28, 33, 36. However, there may be other conditions that maximize the therapeutic effect of chemotherapies combined with US and MBs, and further research aimed at identifying them is needed. In the present study, only one cell (MBT-2, murine bladder cancer cell) line was used in the* in vitro* and* in vivo* experiments. It is necessary to determine whether US irradiation in the presence of MBs can improve the antitumor effect of anticancer drugs against various types of cancer cells, including human bladder cancer.

In the present study, the expression levels of apoptosis-related genes including BAX, bcl-2, p53, caspase3, caspase8, and caspase9 were investigated to determine the underlying mechanisms that explain the results of cell viability measurements *in vitro*. The main mode of action of MMC is the induction of apoptosis in a dose and time-dependent manner 37. In addition, apoptosis is reported to be induced by US irradiation alone or when combined with MBs 38. Antitumor agents combined with MBs and US significantly promoted apoptosis in target cells more effectively than antitumor agents alone, as reported previously 33, 39. In the present study, there was a trend for expression levels of apoptotic-related genes, including BAX, p53, caspase3, caspase8, and caspase9, to be higher in the US + MB + MMC group than in the other three groups, but the differences were not significant (Fig. 2). The results of experiments measuring the expression levels of apoptosis-related genes showed that apoptosis might not be an underlying mechanism explaining the results obtained from *in vitro* cell viability measurements. Other mechanisms, e.g., production of free radicals, might be candidates 40; however, the present study could not identify them.

In the present study, a bladder cancer model was established using C3H/HeN mice and MBT-2 cells in combination with chemical and mechanical damage to the urothelium. This orthotopic bladder cancer model mimics the development of bladder cancer through a mechanism of re-implantation after TURBT. In the present study, whether US irradiation in the presence of MBs contributed to efficient delivery of exogenous molecules to tumors in the bladder was investigated. In an *in vitro* study in which the efficiency of doxorubicin introduction into retinoblastoma cells was investigated, in comparison with doxorubicin administration alone, the combination of US and MBs enhanced the efficiency of the introduction of doxorubicin to these cells 41. In our previous report, fluorescent molecules were introduced into the superficial layer of mouse normal bladder wall (urothelium and subepithelial connective tissue) using US and MBs 26. In the present study, fluorescent molecules were successfully introduced into bladder tumors and a layer of normal mucosa by US and MBs. However, their introduction was limited to the superficial area of the bladder tumor. Their penetration into deep areas of bladder tumors was not shown, and delivery of TOTO-3 fluorophores into areas other than the area shown in Figure 3j was not observed. The results suggested that, although the sonoporation technique potentially enhanced the delivery of exogenous molecules to tumors in the bladder, a single treatment might not be sufficient to deliver them into the deep areas of the bladder tumors. In the present study, whether TOTO-3 could passively diffuse into the tumor cells or surrounding normal tissues in the bladder was not rigorously determined. However, the aforementioned results suggested that TOTO-3 was not taken into them, or, even if TOTO-3 was taken into them, it would be in a very small amount and might not reach the threshold that could be detected with a confocal microscope when US irradiation in the presence with MBs was not performed.

Subsequently, the antitumor effect of intravesical instillation of chemotherapy combined with US and MBs was investigated in an experimental bladder cancer model. In the present study, only one treatment session was performed to evaluate the effectiveness and safety of this strategy (intravesical chemotherapy combined with US and MBs) as a single treatment, and treatment was carried out on day 2, because the minimum time interval between tumor cell inoculation and detection of tumor in the bladder using the HFUS imaging system was two days. The dose of MMC was determined based on a previous report 42. There was only a small difference in normalized tumor volume between the control and US + MB groups throughout the entire research period, suggesting that US combined with MBs did not exert an antitumor effect in this study. There was a trend towards smaller normalized tumor volume obtained on day 10 in the US + MB + MMC group than in the MMC group, but the difference was not significant. These results showed that MBs and US might enhance the anti-tumor effect of intravesical instillation of MMC, but clear superiority over conventional treatment (intravesical chemotherapy alone) was not demonstrated. Preclinical studies have been performed to determine whether the antitumor effect and delivery efficiency of intravesically administered antitumor agents to bladder tumors are enhanced by US irradiation in the presence of nano-microbubbles using an orthostatic bladder cancer model 43, 44. When gemcitabine and MBs were co-administered, US irradiation did not enhance delivery efficiency and the expression level of cleaved caspase3 compared with control 43. However, when MMC and oxygen nanobubbles were used, US irradiation significantly enhanced the antitumor effect of MMC 44. The differences in US parameters and types of nano-microbubbles and antitumor agents may contribute to the conflicting results from these studies. To the best of our knowledge, it has not yet been determined under what conditions (e.g., US parameters and the types of nano-microbubbles) intravesical chemotherapy combined with US and nano-microbubbles exerts the greatest synergistic antitumor effect against bladder tumors.

On examination of fluorescent molecules delivery, US and MBs might enhance delivery of antitumor drugs to the tumor in the bladder, but single treatment was not enough to deliver them to deep areas of the tumor. High-intensity US or US irradiation in the presence of MBs may induce cavitation, which leads to mechanical injury of target tissues and increases their temperature; the severity of the damage caused by cavitation varies based on the tissues/organs, as previously reported 45-47. In the present study, experiments aiming to assess damage to the target and surrounding tissues by the current strategy were not performed. However, visible damage to the surrounding normal tissue (e.g., mucosa and submucosal tissue) was not found on HE-stained sections from any groups, as seen in Figure 4, which suggested that the current strategy could be safely applied for the treatment of bladder cancer. In addition, the strategy can be performed multiple times. Repeating this approach (intravesical chemotherapy combined with MBs and US) may promote tumor shrinkage and improvement in the delivery of chemotherapeutic drugs to the deep areas of tumors in the bladder. To determine the optimal treatment schedule (number of treatments and treatment interval) and US conditions that may increase the antitumor effect of intravesical instillation of MMC combined with US and MBs, more studies are needed. In the present study, a comparison of the antitumor effect was not performed when low doses of MMC were administered intravesically, and then US was applied. If chemotherapeutic agents at low concentrations combined with US and MBs can obtain a satisfactory antitumor effect with fewer adverse events, they would provide a benefit for patients with bladder cancer. Further experiments are necessary to analyze the treatment efficacy of the combinations when a low dosage of MMC is used.

There are limitations to this study. The bladder tumors of the present model grew mainly in the submucosa (Fig. 5), different from the common growth pattern of NMIBC in clinical practice. However, as shown in Figure 4, MMC administered intravesically was thought to be delivered to the tumors in the bladder and contributed to their shrinkage. In the *in vivo* US treatment experiment, longitudinal analyses of tumor volume measured by HFUS and histopathological evaluation (using only HE staining) were performed to evaluate the treatment effects of each therapy for tumor in the bladder. Additional histopathological analysis and molecular biology experiments to assess necrosis, apoptosis, and cell cycle control in the tumor may help identify the mechanisms by which the current strategy exerts its antitumor effect on tumors in the bladder. In the present study, the tumor in the bladder was irradiated by US through the abdominal wall. This is not necessarily the most efficient for irradiating tumors in the bladders of experimental mice. However, if this technique is to be applied clinically, US irradiation is expected to be performed from the ventral side. Thus, this irradiation method was used. In the present study, the dynamics of MBs (Sonazoid) in the bladder of experimental mice when the bladder filled with liquid containing MBs was irradiated by US under the current parameters were not analyzed. Such analysis may help identify the optimal US parameters. The concentrations of antitumor agents and US parameters are closely associated with the antitumor effect when they are combined with US and MBs. US parameters were determined based on our previous research 26, 28, 36. However, additional experiments including analysis of US-driven dynamics of MBs, are necessary to determine the optimal conditions under which the greatest therapeutic effect will be obtained by the current combination therapy.

## Conclusions

US irradiation in the presence of MBs (sonoporation) improves the antitumor effect of chemotherapeutic agents on bladder cancer cells, and it may enhance drug delivery of chemotherapeutic agents to tumors in the bladder and the antitumor activity of intravesical chemotherapy against them. Further studies are needed to evaluate the antitumor effect against bladder tumors when intravesical chemotherapy combined with US and MBs is performed repeatedly and to determine the optimal conditions for maximizing the antitumor action of this combination therapy. In addition, it will be necessary to determine whether US irradiation in the presence of MBs enhances the antitumor activity of anticancer agents against bladder cancer tissues derived from clinical specimens and bladder cancer in clinical practice.

## Abbreviations

BCG: bacillus Calmette-Guérin
DAPI: 4,6-diamidino-2-phenylindole
EORTC: European Organization for Research and Treatment of Cancer
HFUS: High-frequency ultrasound
IPIC: Immediate postoperative instillation of chemotherapy
MBs: Microbubbles
MMC: Mitomycin C
MTT: 3-(4,5-dimethylthiazol-2-yl)-2,5-diphenyltetrazolium bromide
NMIBC: Non-muscle-invasive bladder cancer
TURBT: Transurethral resection of the bladder tumor
US: Ultrasound
